# Thiamine-Functionalized Maleated Chitosan: A Novel Bio-Based Adsorbent for Efficient Uptake of Methylene Blue from Aquatic Solutions

**DOI:** 10.3390/molecules31101553

**Published:** 2026-05-07

**Authors:** Ibrahim Hotan Alsohaimi, Mosaed S. Alhumaimess, Ayoub Abdullah Alqadami, Yasser A. El-Ossaily, Abdullah M. Aldawsari, Hamud A. Altaleb, Hassan M. A. Hassan

**Affiliations:** 1 Department of Chemistry, College of Science, Jouf University, Sakaka P.O. Box 2014, Saudi Arabia; mosaed@ju.edu.sa (M.S.A.); yaboubakr@ju.edu.sa (Y.A.E.-O.); 2 Department of Industrial Chemistry, Faculty of Applied Science, University of Hajjah, Hajjah 1104, Yemen; ayoub.qudami@alraziuni.edu.ye; 3 Department of Pharmacy, Faculty of Medicine and Medical Science, Al-Razi University, Sana’a 1152, Yemen; 4 Department of Chemistry, College of Science and Humanities, Prince Sattam Bin Abdulaziz University, Al-Kharj 16273, Saudi Arabia; abdullah.aldawsari@psau.edu.sa; 5 Department of Chemistry, Faculty of Science, Islamic University of Madinah, Madinah 41477, Saudi Arabia; haltaleb@iu.edu.sa

**Keywords:** adsorption, bio-based adsorbent, chitosan, isotherm, methylene blue, thiamine functionalization

## Abstract

A new type of bio-based adsorbents thiamine-functionalized maleated chitosan (CSMA@TA) was prepared and tested to help the effective removal of methylene blue (MB) in water systems. Successful functionalization was confirmed using structural and surface analysis by FTIR, SEM, XRD, TGA, BET and XPS that revealed a mesoporous structure with a surface area of 50.61 m^2^/g, pore volume of 0.062 cm^3^/g and an average pore diameter of 2.65 nm, as well as incorporation of active sites containing nitrogen and sulfur. The best fit of the Langmuir model (*R*^2^ ≈ 0.986; *RMSE* less than 1.0) demonstrated that the adsorption capacity of CSMA@TA was highly dependent on operation parameters, with an optimum adsorption capacity of about 230 mg/g and a removal efficiency of more than 93.4% under an initial MB concentration of 25 mg/L. Kinetic studies followed the pseudo-second-order model (*R*^2^ ≈ 0.986), indicating that the uptake was dominated by chemisorption. Analysis of intraparticle diffusion indicated that the adsorption process involved three stages: diffusion in the boundary layer (k1d = 17.95 mg/g·min^−1/2^), which controlled the first stage; gradual diffusion in the pore diffusion; and stabilization of the equilibrium. The thermodynamic parameters indicated the presence of strong adsorbate-adsorbent interactions and interfacial structuring. ∆*G*° values ranged between −24.85 and −23.56 kJ/mol, ∆*H*° = −44.08 kJ/mol, and ∆*S*° = −64.65 J/molK indicated strong adsorbate-adsorbent interactions and interfacial structuring. The adsorbent also exhibited good reusability, retaining more than 90% of its initial efficiency after five cycles, making it stable. The enhanced performance of CSMA@TA is due to the synergistic effect of carboxyl groups and heteroaromatic thiamine moieties, which enable electrostatic attraction, hydrogen bonding, and π–π interactions. These findings support the claim that CSMA@TA is a high-efficiency, sustainable, and reusable adsorbent with strong potential for practical wastewater treatment applications.

## 1. Introduction

One of the main environmental issues that should not be overlooked is water pollution caused by synthetic pigments, especially in industries such as paper, plastics, and textiles, where large amounts of dyes are discharged into water sources [1,2,3,4]. The characteristics of the dyes include high resistance to degradation, which extends their lifespan [5,6]. In addition to environmental degradation, long-term exposure to these dyes in humans might have health effects [7,8]. Methylene blue is a widely used dye, primarily in medical and industrial practice. The model dye used in this study was methylene blue (MB) because it is widely used in industry and its environmental impact is well-documented. MB is a cationic dye widely used in the textile, printing, paper, and pharmaceutical industries and is commonly found in industrial wastewater streams. Its high-water solubility, combined with its known toxicity to aquatic organisms and to human health, makes it an important target for removal from polluted water. Analytically, MB also offers advantages, including a well-defined molecular structure, a strong absorbance peak at 665 nm, and good stability in aqueous solutions, all of which are beneficial for quantification and comparison with other studies. However, excessive exposure to this dye can lead to adverse health effects, including skin and respiratory irritation, and in some cases, it may affect the nervous system and blood cells [9,10,11]. Removing methylene blue from water is crucial to preventing environmental and health hazards. Due to its strong chemical stability, this dye can persist in water sources, thereby reducing water quality and negatively impacting aquatic ecosystems [12].

Different techniques were employed to uptake MB dye from contaminated water, including adsorption, chemical processes, biological treatments, photocatalysis, and membrane filtration [13,14,15,16]. Among these approaches, adsorption is considered one of the most effective methods for removing contaminants from water [17]. It is highly powerful, easy to implement, cost-efficient, and environmentally friendly, making it a preferred choice for eliminating methylene blue and other pollutants from aqueous solutions [18]. Various materials have been used to remove methylene blue (MB) from contaminated water, including commercial activated carbon, fly ash, clay minerals, and carbon derived from agricultural residues such as wheat straw and sawdust [19,20]. Many of them still exhibit limited adsorption capacity. To enhance the effectiveness of adsorption-based treatment methods, it is essential to develop readily available, cost-effective adsorbents with improved adsorption performance.

One of the natural biopolymers that has generated considerable interest across medicine, agriculture, and environmental science is chitosan [21]. One of the most notable applications of it is the removal of organic dyes, heavy metals, and other organic pollutants from aqueous environments [22]. This efficiency is attributed to the presence of high-density groups of free amino (–NH_2_) and hydroxyl (–OH) groups on its backbone, which serve as active sites for adsorption, coordination, and complexation with contaminants via chelation, electrostatic attraction, and hydrogen bonding [23]. Moreover, chitosan is characterized by its biodegradability, chemical stability, renewability, non-toxicity, and strong chelating ability, making it an environmentally friendly and effective material for water purification [21]. Nevertheless, the intrinsic disadvantages of native chitosan, including low mechanical strength, low surface area, and poor adsorption selectivity, necessitate chemical modification to improve its performance. To overcome these shortcomings, scientists have considered different forms of chitosan, including grafted, crosslinked, and composite [24]. M, maleated chitosan (CSMA), functionalized with maleic anhydride, introduces carboxylic groups, which increase water solubility and provide additional active sites to adsorb. Subsequent surface modification using functional molecules can enhance its surface reactivity and selectivity to certain pollutants.

In this work, we describe a new thiamine-functionalized maleated chitosan (CSMA@TA) composite as a high-performance bio-adsorbent for removing methylene blue from aqueous solutions. It is the first report on thiamine (vitamin B1), a biologically active, nitrogen- and sulfur-based compound, covalently grafted onto maleated chitosan for use as a water treatment agent [25,26]. Thiamine was chosen as the functionalizing agent in the study because it has unique structural and practical characteristics compared with other bioactive molecules, such as cysteine, glutathione, and thiourea derivatives. In contrast to these alternatives, thiamine has both nitrogen and sulfur heteroatoms built into the pyrimidine and thiazole ring systems, which add several binding functionalities (–NH, –OH, -C=N-, and -C-S-). This structural diversity allows multiple adsorption mechanisms to coexist with adsorption by cationic dyes like methylene blue, thereby increasing overall adsorption efficiency. Furthermore, thiamine is a naturally water-soluble and environmentally benign vitamin (B1); as such, it is more sustainable and non-toxic than some synthetic modifiers, including thiourea derivatives, which may raise environmental or safety concerns. It is also cheaper and readily available in large quantities compared to more complex biomolecules, such as glutathione, and thus is better suited for scalable water treatment applications. Notably, thiamine grafting in this study was coupled with maleation of chitosan to introduce more carboxylic groups, thereby introducing a dual-modification strategy that combinatorially enhanced the adsorption capacity and overall performance of the resulting CSMA@TA composite.

The new aspect of this study is the development of a thiamine-functionalized maleated chitosan composite (CSMA@TA), which, to the best of our knowledge, has not been reported previously for dye adsorption. The new functionalization process presents several active sites by two key functional changes: (i) maleation, which grafts carboxylic functionalities to enhance hydrophilicity and charge density, and (ii) thiamine incorporation, which incorporates nitrogen-rich thiazole rings with the ability to engage in π–π interactions and hydrogen bonding. This two-fold change increases the surface reactivity and selectivity of the composite towards cationic dyes, including methylene blue (MB).

## 2. Experimental Section

### 2.1. Chemicals

Thiamine (vitamin B1; TA, C_12_H_17_N_4_OS, 337.27 g/mol, ≥99%), thionyl chloride (SOCl_2_, 118.97 g/mol, ≥99%), maleic anhydride (MA, C_4_H_2_O_3_, 98.06 g/mol, 99%), sodium hydroxide (40 g/mol, ≥98%), ethanol (C_2_H_6_O, 46.07 g/mol, ≥99.5%) and hydrochloric acid (HCl, 36.46 g/mol, 36.5–38%) were all delivered from Sigma-Aldrich Co. (St. Louis, MO, USA). All reagents were of analytical (AR) grade and were used without further purification.

### 2.2. Instruments

The physicochemical properties of CSMA, CSMA@TA, and MB-loaded CSMA@TA were characterized using multiple techniques. FTIR (Shimadzu IR Tracer-100, Shimadzu, Duisburg, Germany) was used to detect functional groups in the 4000–400 cm^−1^ range (4 cm^−1^ resolution, 32 scans) in ATR mode, without any further sample preparation. XPS (Thermo Scientific K-Alpha, Madrid, Spain) was used to analyze elemental composition and chemical states under a base pressure of less than 1 × 10^−8^ Torr. The ex-amendment of crystalline structure was done using XRD at 2θ = 5–80°. Surface morphology was observed by SEM (Quattro S, Thermo Scientific, Madrid, Spain) at 15 kV after gold sputter coating. Thermal stability was analyzed using TGA (Shimadzu TGA-51, Shimadzu) from 25 to 600 °C in air.

### 2.3. Materials Fabrication

#### 2.3.1. Fabrication of Maleated Chitosan (CSMA)

**Figure 1:**
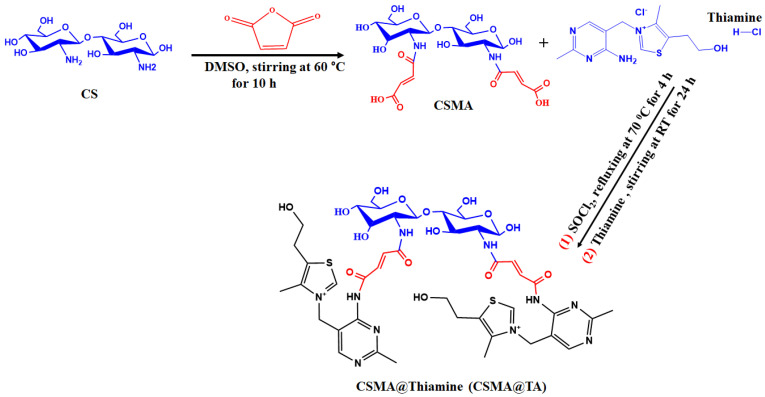
Schematic illustration of the preparation process of poly CSMA@TA.

Maleated chitosan (CSMA) was produced via a simple chemical modification procedure. In short, 1.3 g of chitosan (CS) and 1.8 g of maleic anhydride (MA) were dispersed in 100 mL of dimethyl sulfoxide (DMSO). The grafting of maleic anhydride on the backbone of chitosan was performed by continuously stirring the reaction mixture at 60 °C for 10 h. The reaction product was then precipitated by adding 250 mL of acetone, then repeatedly washing (three times) the reaction product with acetone and ether to remove any unreacted materials or solvent residues. The obtained CSMA was then dried under vacuum to yield the final product (Figure 1).

#### 2.3.2. Synthesis of Maleated Chitosan @Thiamine (CSMA@TA) Composite

The CSMA@TA composite was synthesized via amidation. To activate the carboxyl groups, 1.2 g of CSMA reacted with 15 mL of thionyl chloride (SOCl_2_, 206 mmol) under reflux for 4 h. The product was dried under vacuum, followed by the removal of excess SOCl_2_. In the next step, 1.0 g of thiamine (Sigma-Aldrich) was first dissolved in 50 mL of deionized water and then gradually added to the previously activated CSMA solution. To ensure maximum coupling between thiamine and CSMA, the reaction was stirred continuously under a nitrogen atmosphere at room temperature and allowed to proceed for 24 h. After completion, the solid product was filtered and thoroughly rinsed with multiple washes to de-ionize it and remove any reaction byproducts. Lastly, the purified CSMA@TA was allowed to dry under ambient conditions for 24 h. The schematic diagram of the synthesis process is depicted in Figure 1.

### 2.4. Batch Adsorption Assessment

The effectiveness of CSMA@TA in adsorbing methylene blue (MB) was evaluated in various batch-mode adsorption experiments. The key operational parameters were varied to explore their effects on the adsorption process, including solution pH (2–8), adsorbent dosage (0.01–0.08 g), contact time (2–240 min), initial MB concentration (25–250 mg/L), and temperature (25–45 °C). In a standard adsorption experiment, 0.01 g of CSMA@TA was added to 25 mL of a 25 mg/L MB solution (pH 8.0), and the suspension was stirred at 100 rpm for 180 min. The initial MB concentration of 25 mg/L was chosen as a representative level often used in adsorption studies, falls within the concentration range typically reported for real dye-containing wastewater, and can be used to reliably evaluate adsorption performance under realistic conditions. The treated solution was filtered (0.45 μm), and residual MB was quantified at 665 nm. Adsorption capacity (*q_e_*) and removal efficiency (%R) were calculated using standard equations.

(1) $$q_{e}=(C_{o}-C_{e})\frac{V}{m}$$

(2) $$\%\;adsorption=\frac{C_{o}-C_{e}}{C_{o}}\times 100$$

where *q_e_* represents the adsorption capacity of MB in mg/g, and *C_o_* and *C_e_* (mg/L) correspond to the initial and final MB contents, respectively. V (L) is the solution volume, while m (g) denotes the CSMA@TA mass. All adsorption experiments were performed in triplicate, and the average values are reported with error bars representing the standard deviation.

## 3. Results and Discussion

### 3.1. Structural and Physicochemical Characterization of the Adsorbent

**Figure 2:**
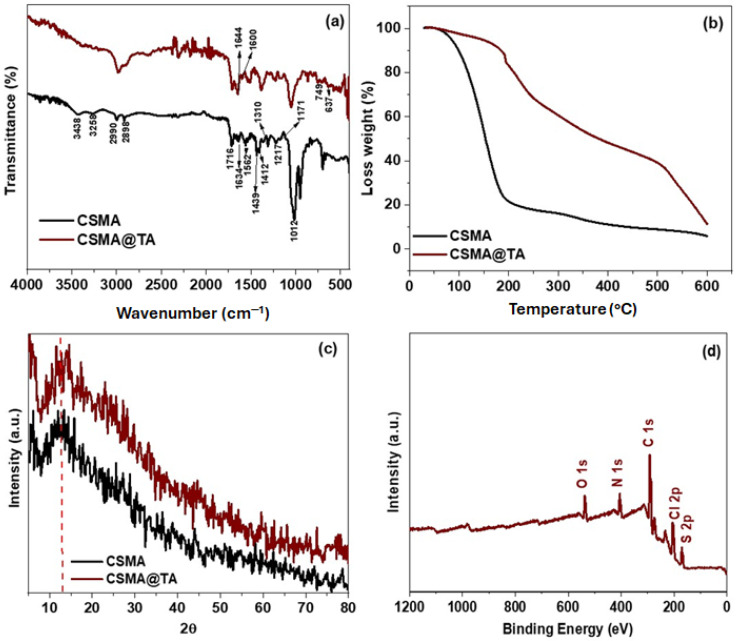
(**a**) FTIR spectra, (**b**) TGA traces, (**c**) XRD patterns of CSMA and CSMA@TA, and (**d**) XPS survey spectrum of CSMA@TA.

The successful preparation of the thiamine-modified maleated chitosan (CSMA@TA) was confirmed by Fourier-transform infrared (FTIR) spectroscopy, which also represented the presence of key functional moieties that promote MB adsorption. These functional groups, introduced through maleation and subsequent thiamine grafting, provide active binding sites, including –COOH, –NH_2_, –C=O, –OH, and heterocyclic rings, which facilitate electrostatic attractions, Hydrogen Bonding, and π–π stacking with methylene blue molecules. As depicted in Figure 2a, the spectra of CSMA and CSMA@TA are presented. The CSMA spectrum displays peaks at 3438, 3258, 2990, 2898, 1716, (1634–1562), (1439–1412), 1310, 1217, and (1171–1012 cm^−1^), which related to the stretching vibrations of overlapping O–H/N–H, asymmetric and symmetric ν(C–H) stretching, C=O stretching in carboxylic acid, -NH-CO, N-H bending, –COO– bending, C–N, C–O, and –C–O–C– stretching vibrations, respectively [27,28,29,30,31,32]. After modifying maleated chitosan with thiamine, the FTIR spectra of CSMA@TA reveal bands at around 1644 cm^−1^ ascribed to the amide II(NH-C=O) stretch [29]. Additionally, the bands at 1600 and 1528 cm^−1^ are attributed to the coupling of υ(C=C) with υ(C=N) stretching vibrations of the pyrimidine ring [33]. The band at ∼749 cm^−1^ is due to the C–S bond. The deformation vibrations of the pyrimidine and triazole [33] are the cause of the bands at ∼637 and ∼749 cm^−1^. The extra bands at 1387, 1237, and 1161 cm^−1^ are associated with the stretching of C–N, C–O, and –C–O–C– bonds, respectively. All these spectral characteristics support the success of the modification of CSMA with thiamine and indicate the presence of numerous functional moieties, including amide, carboxyl, hydroxyl, and heterocyclic rings, which are essential for the effective adsorption of methylene blue through multiple interaction mechanisms.

The thermal stability of the synthesized materials was evaluated by TGA, and the resulting TGA curves for CSMA and CSMA@TA are shown in Figure 2b. The TGA profile of CSMA shows three distinct steps of thermal degradation, with a total weight loss of about 95. The first step, which occurs between 30 and 100 °C, is associated with a loss of about 10 percent of the weight, mainly due to the evaporation of physically adsorbed moisture. The second step, observed between 100 and 210 °C, corresponds to a significant weight loss (~70%) due to the thermal decomposition of low-molecular-weight constituents and functional groups formed by both maleic anhydride (MA) and chitosan (CS) [34]. A further loss of mass, estimated at approximately 15% of the total mass, occurs in the third phase between 210 and 650 °C, which may be due to further breakdown of thermally more stable carbonaceous structures, such as cross-linked chitosan backbones and remaining MA [35,36]. A three-step degradation pattern, however, with strikingly different thermal behavior, can also be observed in the TGA curve of CSMA@TA. The total weight loss of CSMA@TA was about 89%, representing a slight improvement in thermal resistance compared to CSMA. The initial weight loss in the 30–100 °C region was much lower (~3%), indicating reduced hydrophilicity and fewer moisture-absorbing sites, probably due to thiamine grafting. The second degradation stage, between 100 and 450 °C, resulted in a ~54% weight loss, due to the thermal degradation of TA moieties and partial degradation of the CS and MA chains. The third step, at 450–600 °C, involved another 32 percent weight loss, with most of it due to the thermal decomposition of more thermally stable pyrimidine and thiazole ring structures found in thiamine, as well as the remaining carbon skeleton of the polymer. Though CSMA@TA is a little less overall thermally stable than CSMA, this observation can be explained by the fact that the incorporation of thermally labile thiamine groups into the CSMA results in an additional functional group and heteroatoms (e.g., nitrogen and sulfur) that increase adsorption properties but do not support high temperatures. It is noteworthy that the thermal stability of CSMA@TA remains sufficient for the desired application, namely aqueous-phase dye adsorption at ambient conditions. These findings also support the successful chemical incorporation of thiamine into the CSMA structure, as evidenced by the modified thermal decomposition profile.

The crystalline structures of CSMA and CSMA@TA were investigated using XRD, and the resulting patterns are presented in Figure 2c. The XRD diffraction pattern of CSMA displays a broad diffraction peak at 2θ ≈ 12°, which is typically associated with the semi-crystalline structure of hydrated chitosan, reflecting its disordered arrangement and partial crystallinity. Notably, the characteristic peak of native chitosan observed around 2θ ≈ 20.7°, corresponding to its crystalline plane, disappears upon interaction with maleic anhydride (MA) [37]. This disappearance indicates successful grafting of MA onto the chitosan backbone, thereby disrupting the regularity of the polymer chains and reducing its crystallinity. Upon further modification with thiamine, the XRD pattern of CSMA@TA exhibits a marked increase in peak intensities, suggesting a significant change in the material’s crystallinity. This increase is attributed to the incorporation of thiamine, which has a more ordered, crystalline molecular structure than amorphous chitosan. The fact that the CSMA@TA composite was successfully formed and that the FTIR and TGA results indicate that structural modification has occurred confirms the formation of the CSMA@TA composite and supports the findings of FTIR and TGA [38]. The observed changes in the diffraction patterns clearly indicate that chemical modification of chitosan by maleation and subsequent thiamine functionalization significantly affects its structural ordering. These modifications are probably able to enhance adsorptive interactions by making more active sites available and creating more favorable surface properties to bind dye.

The surface elemental composition of the CSMA@TA composite was investigated using X-ray photoelectron spectroscopy (XPS), and the corresponding survey spectrum is displayed in Figure 2d. The spectrum reveals five prominent peaks at binding energies of approximately 290.77 eV (C 1s), 405.19 eV (N 1s), 170.62 eV (O 1s), 537.62 eV (S 2p), and 204.25 eV (Cl 2p). The relative atomic concentrations of these elements were determined to be C 1s (60.88%), N 1s (11.55%), O 1s (8.31%), S 2p (6.81%), and Cl 2p (12.45%). The polysaccharide backbone of chitosan and the aromatic structure of thiamine are reflected by the high carbon content. Nitrogen presence implies the presence of amine and amide functionalities, which are both of chitosan and thiamine. At the same time, the oxygen value is related to the formation of hydroxyl, carboxyl, and carbonyl groups during maleation and amidation. Importantly, incorporation of sulfur (S 2p) and chlorine (Cl 2p) is a direct indicator of thiamine incorporation, as they are not present in native chitosan and maleic anhydride but are an in-built component of the thiazole and pyrimidine rings of thiamine. The successful covalent grafting of thia-mine on the maleated chitosan matrix is confirmed by the presence of these heteroatoms, which are predominated by nitrogen, sulfur, and oxygen. These components also provide active sites that promote electrostatic, hydrogen-bonding, and π–π interactions, which are crucial for MB dye adsorption and further justify the structural modification strategy adopted in this work.

**Figure 3:**
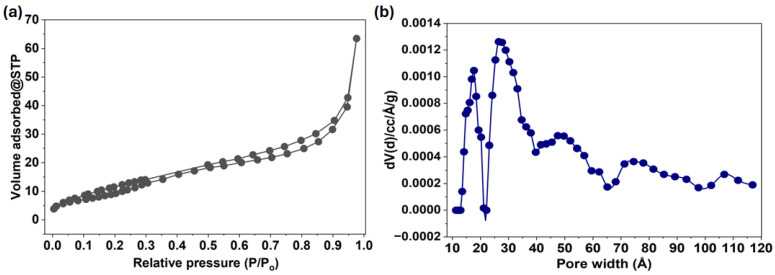
(**a**) N_2_ adsorption–desorption isotherm of CSMA@TA measured at 77 K, and (**b**) the corresponding pore size distribution curve.

N_2_ adsorption-desorption analysis at 77K was used to determine the textural properties of the CSMA@TA composite, as shown in Figure 3a. The isotherm is typical Type IV with a pronounced hysteresis loop, indicating the presence of a mesoporous structure. This observation indicates that capillary condensation occurs within the pores, which is typically observed in materials with well-developed mesoporosity. The gradual increase in adsorption volume at low relative pressure (P/Po < 0.3) is correlated to mono-layer-multilayer adsorption on the surface, whereas the steep increase in adsorption volume at higher relative pressure (P/Po > 0.9) is associated with capillary condensation in larger pores or interparticle vacuoles. Brunauer-Emmett-Teller (BET) analysis showed a specific surface area of 50.61 m^2^/g, which is relatively high for a modified biopolymer-based adsorbent and provides sufficient active sites for dye adsorption. The total pore volume of 0.062 cm^3^/g further confirms the presence of accessible pores that enable the mass transfer of MB molecules into the adsorbent’s internal structure. A dominant peak with a center at approximately 26.47 A confirms that the material is mostly mesoporous, as indicated by the corresponding pore size distribution curve (Figure 3b). Having mesopores would be especially beneficial for the adsorption of comparatively large organic dye molecules, such as methylene blue, since they minimize diffusion resistance and maximize the accessibility of internal adsorption sites. Also, the distribution suggests a mix of uniform mesopores and more extensive pore structures, which may enhance adsorption rates. The moderate surface area, well-developed mesoporosity, and appropriate pore volume suggest that CSMA@TA has a favorable porous structure. These structural characteristics are important for improving adsorption behavior because they promote efficient diffusion, the presence of large numbers of available binding sites, and strong interactions between the adsorbent surface and MB molecules.

**Figure 4:**
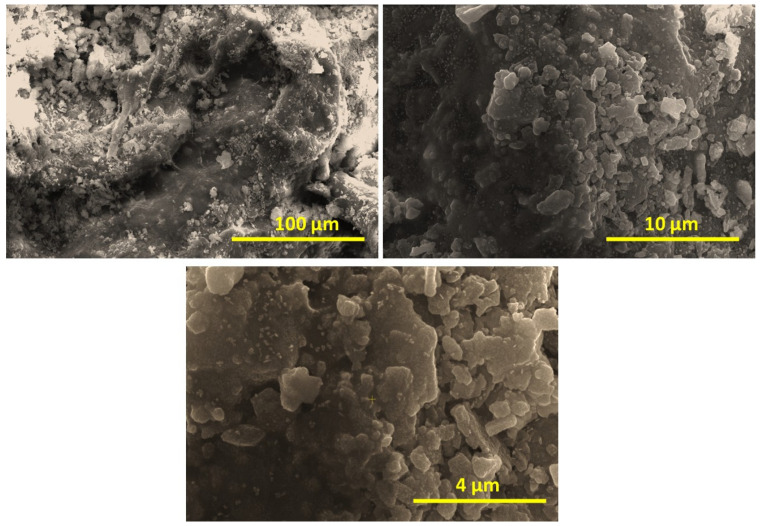
SEM images of the CSMA@TA composite at different magnifications.

SEM images of the CSMA@TA composite are shown in Figure 4, and, as the images were captured at different magnifications, important information regarding both surface morphology and structural texture of the synthesized material was obtained. At lower magnification (top image), the composite has a heterogeneous, rough surface texture with a network of irregularly shaped particles embedded in a semi-continuous matrix. These porous and uneven structures are indicative of the successful incorporation of thiamine into the maleated chitosan backbone, which likely disrupts the smooth surface typically associated with pure chitosan and creates more surface irregularities and greater porosity, both of which are beneficial for dye adsorption. At medium magnification (middle image), the well-dispersed granular structures become more visible. These micro-aggregates are loosely packed and irregular, contributing to a high surface area. The jagged surface can increase the number of accessible active centers and enhance interactions with dye molecules through electrostatic attraction, hydrogen bonding, and π–π stacking. Increasing magnification (bottom image) reveals fine surface features and smaller crystalline-like domains. These micro- and nano-sized particulates exhibit sharp edges and dense packing, which could result from thiamine crystallites or localized molecular ordering within the polymer matrix. The anticipated strong surface interactions between granularity and micro-roughness are expected to contribute to the high adsorption capacity observed in batch experiments. The SEM analysis shows that the CSMA@TA has a morphologically rich, porous surface, which is ideal for adsorptive interactions. The hierarchical harshness and varied particle sizes enhance effective dye diffusion and retention, which justifies the structural changes aimed at boosting adsorptive effectiveness.

### 3.2. Adsorption Performance Evaluation

#### 3.2.1. Influence of Solution pH

**Figure 5:**
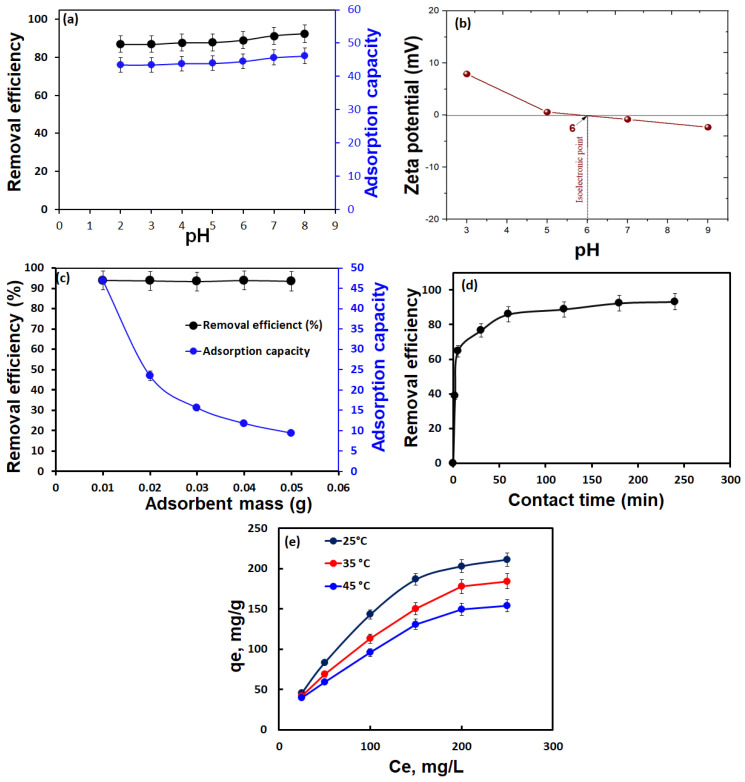
Influence of various parameters on the adsorption of methylene blue (MB) dye onto CSMA@TA: (**a**) solution pH (*C_o_* = 25 mg/L, *T* = 25 °C, volume = 25 mL, time = 1440 min) (**b**) effect of zeta potential, (**c**) adsorbent dosage (same conditions as in (**a**)), (**d**) contact time (*C_o_* = 25 mg/L, *T* = 25 °C, volume = 25 mL, dose = 0.01 g), and (**e**) initial MB concentration (m = 0.01 g, *T* = 25–45 °C, time = 180 min).

The influence of pH on the removal process of MB by CSMA@TA adsorbent was studied over a range of values (pH = 2–8) [(*C_o_* = 25 mg/L; *T* = 25 °C; volume: 25 mL; time = 1440 min)], as shown in Figure 5a. The result depicted that the maximum uptake efficiency of 93.4% was observed at pH 8. This tendency could be attributed to the analysis of the surface charge properties of CSMA@TA, as revealed by zeta potential measurements (Inset of Figure 5a). When the pH of the surface is low (35), there is a strong positive charge on the surface, and the zeta potential is not zero. The condition causes electrostatic repulsion of the cationic MB dye molecules and the protonated functional groups on the adsorbent, as well as competition with excess H^+^ ions for binding sites, thus reducing adsorption efficiency. Still, the high removal efficiency observed even under acidic conditions indicates that other mechanisms, such as hydrogen bonding and π–π interactions, also contribute to MB uptake. The zeta potential curve indicates that the isoelectric point (IEP) of CSMA@TA is around pH 6, where the surface charge is nearly neutral. Beyond this pH (pH > 6), the adsorbent surface becomes increasingly negatively charged as carboxyl and hydroxyl groups deprotonate. This increases the coulombic attraction between the negatively charged adsorbent surface and the positively charged MB molecules, dramatically improving adsorption performance at alkaline pH. CSMA@TA exhibited its maximum removal capacity for MB dye at pH 8, 46.2 mg/g. These results indicate that electrostatic interactions are the dominant mechanism in regulating MB uptake at higher pH values, whereas hydrogen bonding and π–π stacking support adsorption at lower pH values. The overall behavior is consistent with previous research on graphene oxide-modified chitosan reported by Basaleh et al. [39], which confirms that pH significantly influences interactions between surface charge and dye-adsorbent interactions.

#### 3.2.2. Influence of CSMA@TA Dose

The effect of CSMA@TA dosage on the adsorption of methylene blue (MB) was determined by varying the mass of the adsorbent used (between 0.01 and 0.05 g) and keeping the concentration of the dye (*C_o_* = 25 mg/L), volume (25 mL), temperature (25 °C), and contact time (1440 min), as illustrated in Figure 5b. The findings showed that 0.01 g of CSMA@TA had a maximum removal efficiency of 93.45%. A further increase in the dosage of CSMA@TA beyond this point produced only a slight increase in the removal performance, reaching 93.84 percent at 0.05 g, indicating that the system was approaching saturation. This removal efficiency plateau with adsorbent mass implies that most MB molecules were removed by the available active sites at the lowest dosage. This low improvement in dye removal beyond 0.01 g can be explained by the saturation of the active surface sites by MB and a reduced concentration gradient, which reduces the driving force for further adsorption. Interestingly, the amount of MB adsorbed on the adsorbent (*q_e_*), i.e., the amount of adsorbent required to adsorb a specific amount of MB, decreased with increasing adsorbent dose. It is a common phenomenon and is often attributed to particle accumulation at higher doses, which reduces available surface area and hinders access to adsorption sites, thereby reducing per-unit uptake capacity [40,41]. According to these results, 0.01 g of CSMA@TA was determined to be the optimal dosage for further experiments, balancing high removal efficiency and effective utilization of the adsorbent material.

#### 3.2.3. Impact of Adsorption Time

The contact time between the active sites and the MB determines the effectiveness of the MB dye removal using CSMA@TA adsorbent. To investigate this, the effect of equilibrium time, 2–240 min, on the adsorption process was studied in the following conditions: (*C_o_* 25 mg/L, m: 0.01 g, and temperature of 25 °C) as shown in Figure 5c. Their results indicated that removal efficiency, expressed as a percentage (R), increased rapidly with contact time between 2 and 60 min, then more gradually between 60 and 180 min. The high removal of the MB dye by the CSMA@TA adsorbent in the first stage is attributed to the large number of adsorption centers on its surface. After 180 min, the ratio of active centers on the CSMA@TA to the total protein concentration was almost constant until 240 min, presumably due to the saturation of active centers on the CSMA@TA by the active protein concentration. As a result, the highest MB removal efficiency of 93.4% was achieved at 180 min. Hence, the optimal equilibrium time for methylene blue (MB) adsorption onto CSMA@TA was determined to be 180 min.

#### 3.2.4. Influence of Initial MB Concentration and Solution Temperature on Adsorption Behavior

The impact of varying initial methylene blue (MB) concentrations, ranging from 25 to 250 mg/L, on the adsorption performance of CSMA@TA was evaluated under controlled conditions (m: 0.01 g, t: 180 min, temperature: 25–45 °C), as illustrated in Figure 5d. Results showed that the adsorption capacity (*q_e_*) of CSMA@TA increased significantly from 46.1 mg/g to 211.3 mg/g as the original MB concentration increased, indicating that higher dye availability enhances the driving force for mass transfer and promotes greater uptake per unit of adsorbent. However, the highest removal efficiency (92.19%) was observed at the lowest MB concentration (25 mg/L). The number of MB molecules at lower dye concentrations was relatively small compared to the abundance of available active sites on the CSMA@TA surface, allowing for efficient adsorption and resulting in a high removal efficiency. In contrast, as the MB concentration rose, the number of dye molecules exceeded the available adsorption sites, leading to site saturation and a subsequent decline in removal efficiency, despite increased adsorption capacity [42]. This concentration-dependent behavior highlights the dual influence of MB availability: while higher concentrations improve adsorption capacity, they reduce the percentage of dye removed due to limited surface saturation. These findings underscore the importance of optimizing initial dye concentrations for practical applications where either high capacity or high removal efficiency is desired. The effectiveness of chitosan chemical modification was experimentally verified through a combination of structural characterization and performance evaluation. FTIR analysis confirmed the successful introduction of new functional groups associated with thiamine grafting, while XPS provided direct evidence of changes in chemical composition after modification. In particular, the appearance of sulfur (S 2p) signals in the XPS spectrum, which are absent in pristine chitosan and maleated chitosan (CSMA), confirms the successful incorporation of thiamine moieties into the polymer matrix. The detected sulfur content serves as a reliable indicator of the grafting efficiency, reflecting the presence of thiazole groups that contribute additional active sites for adsorption. Furthermore, changes in surface chemistry were supported by zeta potential measurements, which indicated enhanced surface charge after modification. Importantly, the effectiveness of the modification is also demonstrated by the significant improvement in adsorption capacity, increasing from ~10 mg/g for pristine chitosan to ~64 mg/g for CSMA and reaching ~230 mg/g for CSMA@TA. This substantial enhancement highlights the synergistic role of carboxyl groups introduced via maleation and nitrogen/sulfur-containing heterocycles from thiamine grafting, which collectively increase the density of active sites and strengthen adsorbate–adsorbent interactions.

### 3.3. Isotherm and Kinetic Modeling of MB Adsorption

#### 3.3.1. The Adsorption Isotherms vs. Equilibrium Data

**Figure 6:**
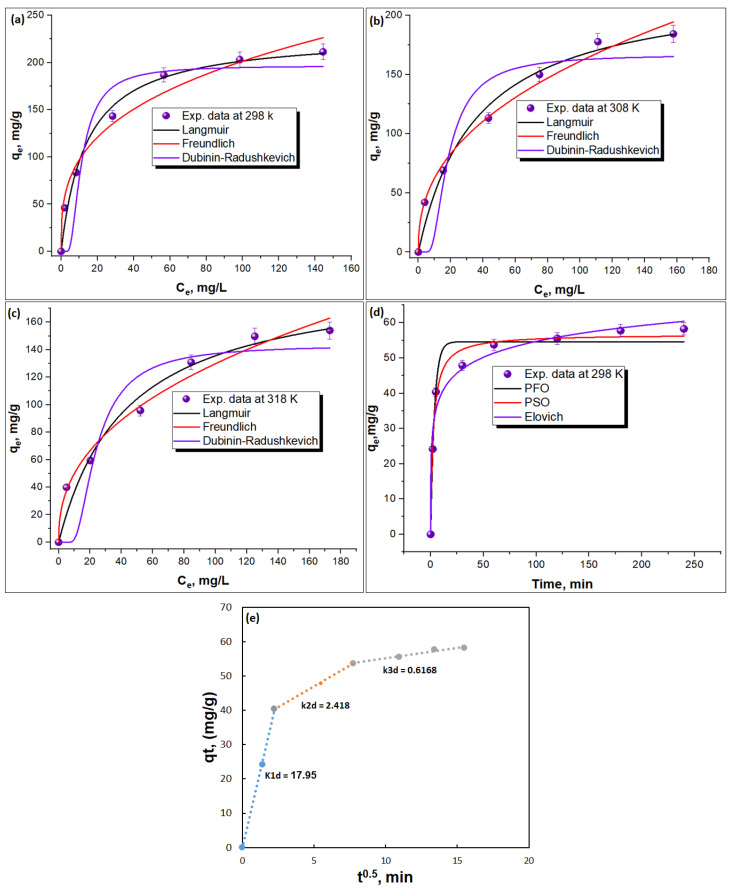
Non-linear isotherm model fittings for methylene blue (MB) adsorption onto CSMA@TA at various temperatures: 25 °C (**a**), 35 °C (**b**), and 45 °C (**c**), demonstrating the temperature-dependent adsorption behavior. (**d**) Non-linear kinetic model fitting illustrating the adsorption kinetics of MB onto CSMA@TA under optimal conditions. (**e**) Intraparticle diffusion model for MB adsorption onto CSMA@TA composite.

The equilibrium data for the adsorption of Methylene blue (MB) onto the CSMA@TA composite were analyzed using three widely known isotherm models, namely: the Langmuir [43,44], the Freundlich [45,46], and the Dubinin-Radushkevich (D-R) models. The fittings of the corresponding nonlinear model are illustrated in Figure 6a–c, and the estimated parameters of the isotherm are summarized in Table 1. The mathematical formulas of these models are provided in the Supplementary Material S1. Of the three isotherm models used, the Langmuir model was the most compatible with the experimental data, with the highest correlation coefficient (*R*^2^ = 0.98555) and the lowest root-mean-square error (*RMSE* = 1.01). Conversely, the Freundlich and Dubinin-Radushkevich (D-R) models exhibited lower fitting performance, with *R*^2^ values of 0.97681 and 0.8671, and *RMSE* values of 4.195 and 5.865, respectively. These findings are consistent with the Langmuir model providing the best explanation of MB adsorption behavior onto CSMA@TA. The higher fit supports the assumption that it is monolayer adsorption on a homogeneously distributed surface of active sites, characteristic of Langmuir-type adsorption processes. The maximum monolayer adsorption capacity (*q_m_*) predicted by the Langmuir model was 230 mg/g, indicating the outstanding affinity of CSMA@TA for MB dye. A comparative analysis of adsorption capacity reveals a gradual increase resulting from chemical modification of chitosan. The pure chitosan (CS) had a relatively low adsorption capacity of about 10 mg/g, which can be attributed to the relatively small number of -NH_2_ and -OH groups available to interact with cationic dye molecules. The introduction of more carboxyl (–COOH) groups on the female counterpart of maleation significantly increased the number of active sites, resulting in an improved adsorption capacity of about 64 mg/g. This shows the positive contribution of carboxyl groups to improved electrostatic interactions with methylene blue. Surprisingly, upon thiamine grafting onto maleated chitosan (CSMA@TA), the adsorption capacity increased exponentially, reaching about 230 mg/g. This significant increase is a clear indication of the synergistic effect of the dual modification strategy: whereas maleation adds carboxyl groups to increase hydrophilicity and charge density, thiamine adds nitrogen- and sulfur-rich heteroaromatic rings that promote additional π–π interactions, hydrogen bonding, and electrostatic interactions. The fact that the dual modification yields a cooperative effect that cannot be obtained with either modification is confirmed by the observation that CSMA to CSMA@TA yields a nearly fourfold improvement compared to CSMA to CSMA and more than 20-fold compared to pristine CSMA. This large adsorption capacity is in stark contrast to that of several reported conventional adsorbents under comparable conditions, such as chitosan/laterite/iron oxide (16 mg/g) [47], nTiO2-Cht/MWCNT (80.65 mg/g) [48], and CuMn_2_O_4_/chitosan (54.05 mg/g) [49]. These comparisons highlight the high efficiency and potential of CSMA@TA as a high-performance adsorbent for dye removal. Moreover, the dimensionless separation factor (R_L_), based on the Langmuir constant, was between 0.3601 and 0.6325, indicating that the adsorption process is favorable (R_L_). Moreover, the Freundlich isotherm model proved insightful, especially the Freundlich constant (n), which was greater than 1. This is the positive, multilayer character of adsorption on a dissimilar surface, which might be strengthened by the diverse functional groups on CSMA@TA. The Langmuir constant (K_L_) and the Freundlich constant (n) were both within the expected range for effective adsorption (01), reaffirming that CSMA@TA has a high affinity for MB molecules and works effectively across a range of concentrations. Taken together, these findings confirm the hypothesis that CSMA@TA is highly adsorptive, owing to the formation of monolayers, very strong dye-adsorbent interactions, and a structurally favorable surface chemistry.

#### 3.3.2. Kinetic Modeling of MB Adsorption onto CSMA@TA

Three widely used kinetic models were used to explain the adsorption mechanism and rate-controlling steps of methylene blue (MB) uptake onto CSMA@TA: the pseudo-first-order, pseudo-second-order, and Elovich models. The results of nonlinear regression analysis are the kinetic parameters given in Table 2, and the fitted curves are obtained in Figure 6d. The mathematical expressions of each model are provided in Supplementary Material S2. Among the three models, the pseudo-second-order model showed the highest agreement with the observed results, as evidenced by the highest *R*^2^ value (0.986). In comparison, the pseudo-first-order and Elovich models yielded slightly lower correlation coefficients (*R*^2^ = 0.972 and 0.974, respectively) than the pseudo-second-order model (*R*^2^ = 0.98578). Moreover, the corresponding root-mean-square error (*RMSE*) values further highlighted the superior fitting performance of the pseudo-second-order model (*RMSE* = 0.97) compared to the pseudo-first-order model (*RMSE* = 3.16). Notably, the adsorption capacity (*q_e_*) predicted by the pseudo-second-order model (56.8 mg/g) was in excellent agreement with the experimentally observed value (57.8 mg/g), further validating the model’s reliability. These results validate the assumption that the adsorption process is primarily controlled by chemisorption, involving valence forces arising from electron sharing or exchange between MB dye molecules and the functional moieties of the CSMA@TA composite. The relatively good fits of the Elovich and pseudo-first-order models also imply, however, that mixed adsorption processes, with both physical adsorption and surface activation, co-exist. To further evaluate the diffusion pathways, the Weber–Morris intraparticle diffusion model was applied (Figure 6e). The resulting plots exhibited three distinct linear regions, corresponding to different diffusion stages, but did not pass through the origin. This confirms that intraparticle diffusion was not the sole rate-limiting step and that boundary-layer effects also contributed significantly. (i) First stage (boundary layer diffusion): This stage was characterized by the steepest slope (k_1_d = 17.95 mg/g·min^−1/2^, *R*^2^ = 0.9985), indicating rapid MB adsorption onto the external surface of CSMA@TA through electrostatic attraction and hydrogen bonding. The sharp increase in adsorption capacity during this stage reflects the abundance of available active sites and strong dye–adsorbent interactions. (ii) Second stage (gradual intraparticle diffusion): The adsorption rate slowed (k_2_d = 2.418 mg/g·min^−1/2^, *R*^2^ = 0.997) as MB molecules began diffusing into the internal pores of CSMA@TA. This stage reflects the progressive occupation of active sites within the adsorbent matrix, with diffusion resistance becoming more pronounced. (iii) Third stage (equilibrium): The slope was lowest (k_3_d = 0.616 mg/g·min^−1/2^, *R*^2^ = 0.972), representing the final equilibrium stage where adsorption slowed considerably due to site saturation and decreased concentration gradients between solution and adsorbent. In sum, the kinetic results indicate that the adsorption of MB onto CSMA@TA is mainly chemisorption-based, yet it also involves multistep diffusion processes. The first rapid uptake occurs due to boundary-layer effects, then to intraparticle diffusion, and finally to equilibrium adsorption. This combination of electrostatic interactions, hydrogen bonding, and π–π stacking is why the CSMA@TA composite has such high efficiency and binding affinity, making it a strong candidate for practical dye removal applications.

#### 3.3.3. Thermodynamics Assessment of MB Uptake

**Figure 7:**
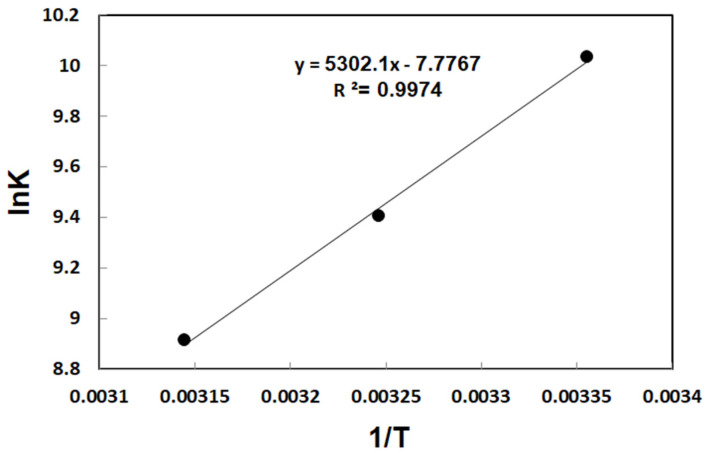
van’t Hoff plot of ln K versus 1/T for the determination of thermodynamic parameters of MB adsorption onto CSMA@TA.

A thermodynamic study was conducted to evaluate the feasibility, spontaneity, and heat change associated with methylene blue (MB) adsorption onto CSMA@TA at 298 K, 308 K, and 318 K. The standard thermodynamic variables, Gibbs free energy change (∆*G*°), enthalpy change (∆*H*°), and entropy change (∆*S*°), were calculated from equilibrium data using established equations. The values of ∆*H*° and ∆*S*° were determined from the slope and intercept, respectively, of the van’t Hoff plot (ln *K_e_*^0^ versus 1/*T*), as illustrated in Figure 7. The linearity of the plot confirms the thermodynamic consistency of the adsorption process. The measured thermodynamic variables are tabulated in Table 3.

(3) $$\ln K_{e}^{0}=-\frac{∆H{}^{\circ}}{RT}+\frac{∆S{}^{\circ}}{R}$$

(4) $$∆G{}^{\circ}=-RT\;\ln K_{e}^{0}$$

(5) $$K_{e}^{0}=\frac{(1000\cdot K_{L}\;molecular\;weight\;of\;adsorbate)\cdot [adsorbate]{}^{\circ}}{\gamma }$$

In this context, $K_{e}^{0}$ represents the dimensionless equilibrium constant. In the thermodynamic calculations, R is the universal gas constant, [adsorbate]° is the standard concentration (1.0 mol L^−1^), and $K_{L}$ is the Langmuir constant. The activity coefficient γ was assumed to be approximately unity under the system’s dilute conditions; therefore, it was not explicitly calculated. Accordingly, the equilibrium constant used in Figure 7 corresponds to $K_{e}^{0}$, derived from the Langmuir constant after appropriate normalization to ensure a dimensionless form.

Table 3 presents the thermodynamic parameters, including enthalpy change (∆*H°*), entropy change (∆*S*°), and Gibbs free energy change (∆*G°*), for the adsorption of methylene blue (MB) onto CSMA@TA at temperatures of 298, 308, and 318 K. The thermodynamic parameters calculated for MB adsorption onto CSMA@TA provide important insights into the underlying mechanism. The adsorption process is exothermic, so the negative enthalpy change (∆*H*° = −44.08 kJ/mol) demonstrates. This is why the adsorption capacity decreases slightly at higher temperatures: higher thermal energy counteracts the exothermic binding process. The Gibbs free energy values (∆*G*°) remained consistently negative across all studied temperatures (−24.85 kJ/mol at 298 K, −24.08 kJ/mol at 308 K, and −23.56 kJ/mol at 318 K), confirming that the adsorption of MB onto CSMA@TA is spontaneous. The reduction in ∆*G*° magnitude with increasing temperature further supports the exothermic nature of the process, in which lower temperatures favor spontaneity. Most notably, the negative entropy change (∆*S*° = −64.65 J/mol·K) reflects a net decrease in the degrees of freedom of the system during adsorption; however, it should not be interpreted solely as a direct measure of reduced “randomness.” In adsorption processes, the overall entropy change arises from a balance between solute immobilization and solvent reorganization. The ability of the MB molecules to bind to the CSMA@TA surface inhibits their translational and rotational motility, which contributes to a negative entropy term. Meanwhile, interactions, including electrostatic attraction, hydrogen bonding, and π–π stacking, facilitate the development of a more organized interfacial structure. The displacement of interfacial water molecules may locally increase the solvent entropy, but this effect seems to be overwhelmed by the ordering associated with dye adsorption and surface complexation. Consequently, the negative ∆*S* observed can be explained by strong adsorbate-adsorbent interfaces and interfacial-structuring effects, rather than by chance or a simple mixing effect. In sum, the negative ∆*H*°, ∆*S*°, and ∆*G*° values all indicate that the adsorption of MB onto CSMA@TA is spontaneous, exothermic, and enthalpy-driven, with the decrease in entropy indicative of the more structured arrangement of dye molecules and the reorganization of the solvent at the adsorbent interface.

### 3.4. Proposed Adsorption Mechanism of MB onto CSMA@TA

**Figure 8:**
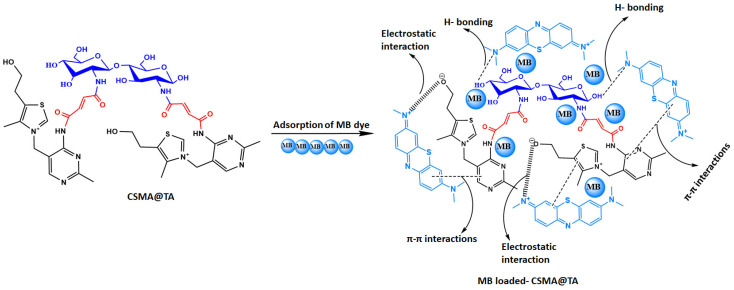
Proposed adsorption mechanism of methylene blue (MB) onto the CSMA@TA composite, illustrating the synergistic interactions, including electrostatic attraction, π–π stacking, hydrogen bonding, and surface complexation, which contribute to the high adsorption efficiency and stability of the material.

Figure 8 shows that the mechanism of MB uptake onto the CSMA@TA composite is a combination of physicochemical interactions arising from the composite’s rich surface functionality. The CSMA@TA surface is functionalized with the hydroxyl (-OH), amine (-NH), carboxyl (-COOH), and thiazole rings, respectively, and is based on the chitosan backbone, maleic anhydride functionalization, and thiamine functionalization, respectively. Under the condition of a pH of 8 (experimentally determined as the optimum condition to remove MB), the surface of CSMA@TA becomes negatively charged with the deprotonation of carboxyl and hydroxyl groups. Such a charge state greatly increases the electrostatic attraction between the positively charged sites on the MB molecule and the negatively charged sites on the CSMA surface covered with the TA. The main driving force behind the rapid and vigorous uptake of MB in aqueous solution is this interaction. In addition to electrostatic forces, the adsorption process is further stabilized by π–π stacking between the aromatic rings of MB and the conjugated heterocyclic systems (thiazole and pyrimidine rings) in the CSMA@TA framework. These π–π interactions increase the selectivity and affinity of the aromatic dye molecules to the adsorbent, as indicated by the high adsorbent capacity (229.88 mg/g) obtained after modeling the Langmuir isotherm. Besides, hydrogen bonding plays a significant role in the adsorption process. In particular, the amino and hydroxyl groups on the surface of the CSMA@TA can form hydrogen bonds with the nitrogen atoms or electron-rich centers on the MB molecule, thereby supporting the overall dye-adsorbent interaction. The multiple bonding interactions above produce a synergistic effect, leading to high efficiency and high binding stability. The Langmuir isotherm model best fits the equilibrium data (*R*^2^ = 0.98555), fully supporting the proposed mechanism, which suggests monolayer adsorption on a homogeneous distribution of active sites. This means that the MB molecules bind to certain, well-defined functional groups on the CSMA@TA surface. Moreover, the kinetic data are in agreement with the pseudo-second-order model (*R*^2^ = 0.98578), indicating that the adsorption process is predominantly chemisorption, driven by valence forces involving the exchange or sharing of electrons. This promotes the multimodal mechanistic pathway of hydrogen bonding and π–π interactions, which is characteristic of chemisorptive processes. The combination of these interaction processes leads to the overall performance of the composite, contributing to high adsorption efficiency, strong affinity for target dyes, and structural stability, making it an attractive candidate for high-performance wastewater dye remediation.

**Figure 9:**
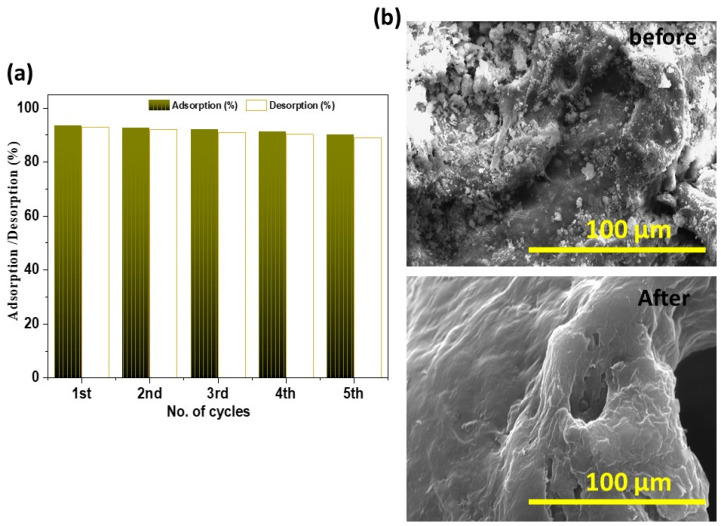
(**a**) Reusability of CSMA@TA for MB adsorption with corresponding (**b**) SEM images before and after reuse.

The reusability of the CSMA@TA adsorbent was evaluated over five consecutive adsorption-desorption cycles, as illustrated in Figure 9a. The adsorption efficiency of the composite in the first cycle was 93.4%, with a desorption recovery of 93%, reaffirming the composite’s high initial stability and regeneration capacity. As the cycle count increased, only a minor decrease in performance was observed. In the second cycle, the adsorption and desorption efficiencies had slightly reduced to 92.5% and 92%, respectively. The adsorption efficiency after five cycles was 90%, with a desorption efficiency of 89%, representing a reduction of less than 5 percent overall compared to the first cycle. This slight decrease is likely due to partial blockage of active sites and possible surface contamination by residual MB units that were not fully eliminated during regeneration. These findings are further supported by the SEM micrographs in Figure 9b, which further reveal the presence of amoebas in the samples. Prior to reuse, the surface of CSMA@TA exhibits a rough, heterogeneous morphology with numerous adsorption sites. The repeated adsorption-desorption cycles result in the surface appearing smoother and slightly compacted, indicating slight structural changes, but the overall integrity of the adsorbent is maintained. All these findings indicate that CSMA@TA has high stability and reusability, with adsorption efficiency exceeding 90% even after 5 cycles. This performance is comparable to that of other chitosan-based adsorbents reported in the literature, which often show significant decreases in efficacy after repeated reuse. The results indicate that the material could be used as a sustainable and low-cost water treatment system, where recyclability is a key parameter.

### 3.5. Comparative Studies

Table 4 provides a comparative summary of the adsorption performance of the recently synthesized CSMA@TA composite with that of a list of previously reported chitosan-based adsorbents for MB removal [50,51,52,53,54,55]. The outcomes clearly demonstrate CSMA@TA’s dominance over its rivals in adsorption capacity, working pH, and equilibrium contact time. CSMA@TA had the highest adsorption capacity of 230 mg/g, compared with other adsorbents listed in the table. An example is the very low adsorption capacity of 7.25 mg/g for pure chitosan (CS) particles, which reflects the number of active sites on pure CS. Likewise, a chitosan/Fe_3_O_4_ nanocomposite showed a modest uptake of 45.4 mg/g, whereas the used coffee/chitosan composite had 75.8 mg/g. Even more advanced additions, such as CS/AC (11.5 mg/g) or UiO-66-NH_2_/guanidine-chitosan (179 mg/g), were less effective than CSMA@TA. Only the similar system, S-CS-MT, reached 188 mg/g, though, again, it did not exceed the performance of CSMA@TA. This indicates that dual functionalization of chitosan with maleic anhydride and thiamine is highly effective in generating new active sites and enhancing binding interactions, thereby greatly improving adsorption performance. Most chitosan-based adsorbents, such as CSMA@TA, were identified to adsorb best under alkaline conditions (pH = 8). This is due to the deprotonation of the carboxyl and hydroxyl groups, thereby increasing electrostatic interactions with the cationic MB dye. In comparison, the spent coffee/chitosan composite adsorbents were most effective at acidic pH (pH = 3), but with lower adsorption efficiency. This makes CSMA@TA more applicable to actual wastewater applications, where alkaline effluents (e.g., in textile industries) are typical. Another important parameter for evaluating the adsorbent’s efficiency is the equilibrium contact time. The equilibrium in CSMA@TA took 180 min, which is quite long compared to UiO-66-NH_2_/guanidine-chitosan (40 min) and S-CS-MT (75 min). But this increased contact time is offset by the material’s increased adsorption capacity and stability. It is also possible that the slower kinetics are due to multilayer diffusion or to strong chemisorption via hydrogen bonding, pi-pi interactions, and electrostatic attraction, as supported by the kinetic and mechanistic analyses carried out in this study. The comparative results show that although some composites (e.g., UiO-66-NH_2_/guanidine–chitosan) have high adsorption capacity, the most suitable working pH is alkaline, and the interaction mechanisms are very robust. This combination of adsorption capacity and environmental compatibility underscores the high potential of CSMA@TA as a viable, sustainable adsorbent for dye removal applications.

### 3.6. Feasibility Study

The viability of using CSMA@TA as a bio-based adsorbent for MB dye removal was substantiated across various dimensions. The CSMA@TA synthesis is technically simple, uses mild reaction conditions, and employs readily available equipment, making it scalable. The preparation is based on a large volume of inexpensive raw materials, including chitosan and thiamine, which keep the estimated production cost at $0.22–$0.30 per gram, making it economically competitive with commercial adsorbent materials, such as activated carbon. The process is also benign for the environment, as it does not use any toxic solvents; it uses biodegradable, non-toxic compounds, thereby complying with the principles of green chemistry and reducing its environmental footprint. Operationally, the adsorbent worked efficiently at normal conditions (pH 8.0, 180 min, 0.01 g dose at 298 K), which are compatible with normal industrial wastewater streams, particularly those of textile effluents. The large adsorption capacity (230 mg/g), good kinetics, and thermodynamics also serve as proof of its practicability. All these aspects confirm the high viability of the CSMA@TA implementation for real-life dye remediation, but further optimization of the CSMA@TA implementation in continuous-flow systems and multi-contaminant matrices remains pending.

### 3.7. Limitations and Future Feasibility

Although the study has performed well in controlled laboratory environments, one notable weakness is that it has not yet examined CSMA@TA’s performance on wastewater samples in real-world settings. Real effluents may have several dyes, salts, and competing contaminants that may influence performance. Also, although the adsorption time is reasonable in laboratory tests, it may need further optimization for real-time use, where faster kinetics are desired. It should also be remembered that some of the best conditions found in this study, especially the dosage of adsorbent (0.01 g) and pH (8), are at the extremes of the ranges studied, and a broader range of operational parameters (e.g., lower dosages or higher pH values) could have given a more detailed picture of how the system works. An extensive analysis of the feasibility of regeneration, life-cycle analysis, and the techno-economic model should also be carried out at the pilot and industrial levels. However, the current work provides strong evidence of concept, and follow-up feasibility studies are recommended to demonstrate the adsorbent’s performance and cost-effectiveness in real-world applications.

## 4. Conclusions

This paper synthesized and used a new bio-functionalized CSMA@TA adsorbent to effectively remove methylene blue (MB) dye from aqueous media. FT-IR, SEM, TGA, XRD, and XPS analyses were used to thoroughly feature the structural and morphological features of the composite. Under optimal conditions (m: 0.01 g, pH: 8.0, t: 180 min, T: 298 K, and agitation speed: 100 rpm), CSMA@TA has shown a maximum adsorption capacity of 230 mg/g. Kinetic and equilibrium studies revealed that the adsorption process was controlled by the Langmuir isotherm model, indicating monolayer adsorption, and conformed to the pseudo-second-order kinetic model, suggesting chemisorption as the dominant mechanism. Analysis based on thermodynamic principles further revealed that the adsorption process was spontaneous and exothermic. The adsorption mechanism was driven by electrostatic interactions, hydrogen bonding, and π–π stacking between the functional groups of CSMA@TA and the MB dye molecules. Altogether, the findings demonstrate that CSMA@TA is a highly effective and promising adsorbent for removing cationic dyes from contaminated water, with a high adsorption capacity and strong interaction mechanisms.

## Figures and Tables

**Table 1:** The isotherm model parameters describing the adsorption behavior of MB onto CSMA@TA composite.

Model	MB		
	298 K	308 K	318 K
***Langmuir***			
*q_m_*, mg/g	230	226	194
*K_L_* (L/mg)	0.07108	0.02797	0.02324
*R_L_*	0.3601	0.5884	0.6325
*R* ^2^	0.98555	0.98909	0.98265
*RMSE*	1.01	0.894	0.892
***Freundlich***			
*K_f_*, (mg/g) (L/mg)^1/n^	46.68	24.180	19.21
*n*	3.15	2.42	2.41
*R* ^2^	0.97681	0.97819	0.96933
*RMSE*	4.195	2.280	2.236
***Dubinin-R***			
*q_s_*, mg/g	197	167	143
*K_D-R_* (mol^2^ kJ^−2^)	41.44	104.36	171.54
*E* (kJ mol^−1^)	0.1098	0.0692	0.0539
*R* ^2^	0.8671	0.8572	0.84873
*RMSE*	5.865	9.154	9.01

**Table 2:** The kinetic model parameters for MB adsorption onto CSMA@TA composite.

*C*_*o*_ (mg/L)	*q*_*e*,*exp.*_ (mg/g)	*Pseudo-First-Order*			*Pseudo-Second-Order*			*Elovich*		
		*q*_*e*1,*cal*._ (mg/g)	*K*_1_ (1/min)	*R*^2^/*RMSE*	*q*_*e*2,*cal*._ (mg/g)	*K*_2_ (g/mg min)	*R* ^2^	*A* (mg/g min)	*B* (mg/g)	*R* ^2^
25	57.8	54.6	0.2792	0.972/3.16	56.8	0.00703	0.986/0.97	296	0.154	0.974

**Table 3:** Thermodynamic parameters describing the spontaneity and nature of MB adsorption onto CSMA@TA over the studied temperature range.

Dye	∆*H*° (kJ/mol)	(−) ∆*S*° (J/mol·K)	∆*G*° (kJ/mol)		
			298 K	308 K	318 K
MB	−44.081	64.65	−24.85	−24.08	−23.56

**Table 4:** Comparison of CSMA@TA with Other Chitosan-Based Adsorbents for MB Removal.

Adsorbent	Adsorption Capacity (mg/g)	Optimal pH	Contact Time (min)	References
CS particles	7.25	6	40	[50]
Chitosan/Fe_3_O_4_ nanocomposite	45.4	9	90	[51]
Spent coffee/chitosan composite	75.8	3	100	[52]
CS/AC	11.5	7	60	[53]
UiO-66-NH_2_/guanidine-chitosan	179	8	40	[54]
S-CS-MT	188	8	75	[55]
CSMA@TA	230	8	180	This work

## Data Availability

The original contributions presented in this study are included in the article/Supplementary Materials. Further inquiries can be directed to the corresponding authors.

## Supplementary Materials

The following supporting information can be downloaded at: https://www.mdpi.com/article/10.3390/molecules31101553/s1, S1: non-linear models; S2: the equation of the pseudo-first-order, pseudo-second-order and Elovich models.
