# Supplementary material for: Thiamine-Functionalized Maleated Chitosan: A Novel Bio-Based Adsorbent for Efficient Uptake of Methylene Blue from Aquatic Solutions
Source: Molecules. 2026 May 7;31(10):1553. doi: 10.3390/molecules31101553 (PMC13209739; doi:10.3390/molecules31101553)
Supplement: Supplementary file 1 [file molecules-31-01553-s001.zip › molecules-4261007-supplementary.pdf]

Supplementary Material

Thiamine-Functionalized Maleated Chitosan: A Novel Bio-Based Adsorbent for Efficient Uptake of Methylene Blue from Aquatic Solutions

Ibrahim Hotan Alsohaimi [1,*], Mosaed S. Alhumaimess [1], Ayoub Abdullah Alqadami [2,3], Yasser A. El-Ossaily [1], Abdullah M. Aldawsari [4], Hamud A. Altaleb [5] and Hassan M. A. Hassan [1,*]

[1] Department of Chemistry, College of Science, Jouf University, Sakaka P.O. Box 2014, Saudi Arabia

[2] Department of Industrial Chemistry, Faculty of Applied Science, University of Hajjah, Hajjah 1104, Yemen

[3] Department of Pharmacy, Faculty of Medicine and Medical Science, Al-Razi University, Sana'a 1152, Yemen

[4] Department of Chemistry, College of Science and Humanities, Prince Sattam Bin Abdulaziz University, Al-Kharj 16273, Saudi Arabia

[5] Department of Chemistry, Faculty of Science, Islamic University of Madinah, Madinah 41477, Saudi Arabia

* Correspondence: ehalshaimi@ju.edu.sa (I.H.A.); hmahmed@ju.edu.sa (H.M.A.H.); Tel.: +966-504-904-183 (I.H.A.); +966-545-119-381 (H.M.A.H.)

Table of contents

S1	Adsorption isotherms
S2	Adsorption kinetics

S1. Adsorption Isotherms

The experimental data were analyzed using non-linear models, including Langmuir[1], Freundlich[2], and Dubinin-Radushkevich (D-R) models. The corresponding equations for these models are provided below.:

$$q_e = \frac{q_m K_L C_e}{1 + K_L C_e} \quad (1)$$

$$q_e = K_F\, {C_e}^{1/n} \quad (2)$$

$$q_e = q_s\, e^{-K_{D-R}\,\varepsilon^2} \quad (3)$$

$$\varepsilon = RTln(1 + \frac{1}{C_e}) \quad (4)$$

$$E = \frac{1}{\sqrt{2K_{D-R}}} \quad (5)$$

where q_e (mg/g) is the amount of MB adsorbed on CSMA@TA at equilibrium, q_m is the maximum adsorption capacity, q_s (mg/g) is the D–R constant. K_L (L/mg) is the Langmuir constant, K_F is the Freundlich constant, and n is the adsorption intensity**.**

S2. Adsorption Kinetics

The equation of the pseudo-first-order (PFO) and pseudo-second-order (PSO)and Elovich models are given below:

$$q_t = q_e(1 - e^{-k_1 t}) \quad (6)$$

$$q_t = \frac{q_e^2 k_2 t}{1 + q_e k_2\, t} \quad (7)$$

$$q_t = \frac{1}{\beta}\ln(1 + \alpha\beta t) \quad (8)$$

where qe and qt (mg/g) are the adsorption capacity at equilibrium and the specific contact time t, respectively. k1 (min^{-1}) and k2 ($g{\cdot}mg^{-1}min^{-1}$) are the rate constant in PFO and PSO kinetic models, respectively. β and α are the desorption rate and adsorption rate, respectively. To better understand the possibility of different mechanisms controlling successive stages of adsorption, the intra-particle diffusion model (Eq. 9) was applied.

$$qt = ki\, t^{0.5} + \mathrm{C} \quad (9)$$

were ki represents the diffusion rate constant ($mg/g{\cdot}min^{-0.5}$), while **C** denotes a constant associated with the thickness of the boundary layer (mg/g).

References:

[1] A. Wallis, M.F. Dollard, Local and global factors in work stress - The Australian dairy farming examplar, Scand J Work Environ Heal Suppl. (2008) 66–74.

[2] Freundlich, H., Ueber Kolloidfällung und Adsorption. Zeitschr f Chem und Ind der Kolloide 1, 321–331 (1907). https://doi.org/10.1007/BF01813604.
